# Sulfur vacancies on MoS_2_ enhanced the activation of peroxymonosulfate through the co-existence of radical and non-radical pathways to degrade organic pollutants in wastewater†

**DOI:** 10.1039/d2ra02448a

**Published:** 2022-09-20

**Authors:** Cai-Wu Luo, Lei Cai, Chao Xie, Gang Li, Tian-Jiao Jiang

**Affiliations:** State Key Laboratory of Safety and Health for Metal Mines, Sinosteel Maanshan General Institute of Mining Research Co., Ltd 243000 China luocaiwu00@126.com +86-734-8282345; Fujian Provincial Key Lab of Coastal Basin Environment, Fujian Polytechnic Normal University 350300 China; School of Resource Environmental and Safety Engineering, University of South China 421000 China

## Abstract

The enhancement of vacancies in catalysts involving Fenton-like reactions is a promising way to remove organic pollutants in wastewater, but sulfur vacancies are rarely involved. In this work, MoS_2_ containing defect sites were synthesized by a simple high-temperature treatment and then applied for activating peroxymonosulfate to eliminate organic pollutants in wastewater. The structure was characterized by several techniques such as XRD, BET, and XPS. Important influencing factors were systemically investigated. The results indicated that MoS_2_ with sulfur vacancies possessed a higher catalytic activity than that of the parent MoS_2_. The annealing temperature of the catalyst had a great effect on the removal of organic pollutants. Besides, the catalytic system had a wide pH range. Quenching and electron paramagnetic resonance (EPR) experiments indicated that the reaction system contained radical and non-radical species. The characterization results revealed that the defect sites in catalysts mainly strengthened the activity of catalysts. This study offers a new heterogeneous catalyst for the removal of organic pollutants *via* the peroxymonosulfate-based Fenton-like reactions.

## Abstract 2

Sulfur vacancies on MoS2 enhanced the activation of peroxymonosulfate to remove organic pollutants in wastewater.

## Graphical abstract

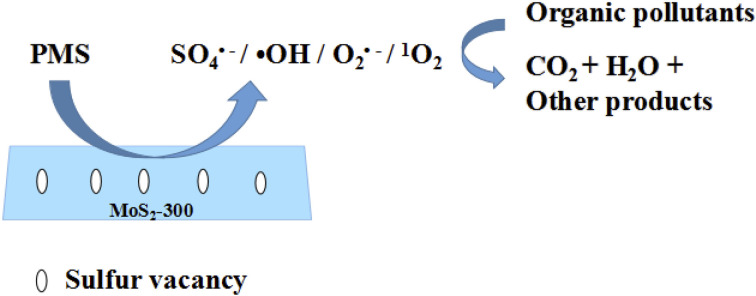

## 1. Introduction

With the development of industries, a large number of organic pollutants are discharged into the water environment, causing a serious problem of water pollution. Many useful technologies including adsorption,^1^ membranes,^2^ and advanced oxidation reactions (AORs)^3–5^ have been developed to dispel organic pollutants. In these ways, the AOR involving peroxymonosulfate (denoted as PMS) as an oxidant is an ideal choice. However, the direct oxidation of organic pollutants by PMS is extremely difficult. Accordingly, it has to introduce external activation factors including heat,^6^ alkaline conditions,^7^ and homogeneous^8,9^ and heterogeneous catalysts.^3–5,10–15^ Among them, a heterogeneous catalyst for the activation of PMS is a good strategy for repeated use, realization of radical and non-radical routes, *etc.* The activation of PMS by heterogeneous catalysts containing defect sites has attracted much attention in the past few years.^16–34^ Among them, the oxygen vacancies are the most studied,^16,18–25^ which often appear in various catalysts containing oxygen, including pure metal oxides such as Co_3_O_4_^16^ and ZnO,^19^ perovskite^20^ and composited compounds.^21–23^ For example, Zhao *et al.*^16^ reported the role of oxygen vacancies in different Co_3_O_4_ crystal planes for the activation of PMS by the formation of ^1^O_2_; Gao *et al.*^20^ demonstrated the correlation between oxygen-deficient sites in the perovskite and the formation of ^1^O_2_ in activated PMS. However, this vacancy was easy to be restored under the aerobic condition. Nitrogen defect sites are the other type of vacancy used for activating PMS.^24,25^ It often appeared the cases with nitrogen-doped carbon materials. For instance, Wan *et al.*^24^ reported the degradation of 4-chlorophenol by N-doped biological carbon-activated PMS. Compared to the nitrogen dopant, the nitrogen vacancy has more affinity with PMS to form a surface polymer. However, the existence of nitrogen vacancies may make the catalyst vulnerable to the attack by reactive oxygen species, resulting in the poor stability of catalyst. Sulfur vacancies used for activating PMS have attracted great attention in recent years.^26–31^ This case is common in transition metal sulfides. Compared to oxygen and nitrogen vacancies, sulfur vacancies possess more advantages, as it is not only that the valence-changing transition metal ions are usually good activators for PMS but also that the sulfur defect sites themselves can activate both PMS and metal ions. Among them, MoS_2_ is an ideal catalyst due to its good ability to activate PMS.^32–34^ Besides, it is responsive to light illumination.^35,36^ It has been reported that introducing vacancies in MoS_2_ enhanced the removal ability towards organic pollutants in the presence of PMS.^30,31^ For example, MoS_2_ containing defect sites was prepared by a mechanical grinding method,^30^ and tetracycline was degraded by the above-mentioned catalyst-activated PMS. In this way, the characteristics of MoS_2_ were optimized, forming sulfur vacancies and exposing more Mo(iv) species. These changes were of great help to degrade tetracycline. However, the promotion was limited. There is no doubt that the development of a novel and effective MoS_2_-based catalyst with vacancies is highly desirable.

In this work, MoS_2_ containing sulfur vacancies was synthesized by a high-temperature treatment to activate PMS and then used to degrade RhB in wastewater. Various influencing factors were investigated. The correlation between the structure of the catalyst and the catalytic activity was discussed.

## 2. Experiments

### 2.1. Chemicals and materials

Various chemicals and materials such as PMS, peroxydisulfate (PDS), hydrogen peroxide (H_2_O_2_, 30 wt%), MoS_2_ powder, rhodamine B, 5,5-Dimethyl-1-pyrroline *N*-oxide (DMPO), *tert*-butanol, tiron, and 4-hydroxy-2,2,6,6-tetramethylpiperidine (TEMP) were purchased from commercial sources. All the above-mentioned chemicals were of analytical grade and employed without further purification.

### 2.2. Synthesis of catalysts

A series of MoS_2_ catalysts containing vacancies were synthesized by heat treatment of MoS_2_ at different temperatures (200, 300, 400 and 500 °C) for different time periods (4, 8 and 12 h) in air atmosphere. They were respectively denoted as MoS_2_-200, MoS_2_-300, MoS_2_-400 and MoS_2_-500. The original powders were not treated by heat, which was denoted as MoS_2_. Unless otherwise stated, the treatment time was 4 h. The preparation process of the catalyst is shown in Fig. S1.†

### 2.3. Characterization of catalysts

The crystal phases of the catalyst were analyzed by X-ray diffraction (XRD) using a D8-Advance X-ray diffractometer (Bruker Corp., Billerica, MA, USA). The specific surface area and pore features of the catalyst were measured by liquid nitrogen physisorption using an Autosorb-iQ/ASAP 2460 apparatus. The morphology of the catalyst was examined by scanning electron microscopy (SEM) using an SU 8100 instrument (Japan). Raman spectra were recorded using a LabRAM HR800. The surface elemental information of catalyst was acquired by X-ray photoelectron spectroscopy (XPS) using an EscaLab 250xi equipment. The concentration of elements in the solution after the reaction was detected by inductively coupled plasma-mass spectrometry (ICP-MS) using an ICP-MS-7700 (Agilent Technologies Co. Ltd., USA). The vacancy of the catalyst and reactive oxygen species were measured by EPR using a Bruker A300.

### 2.4. Degradation of organic pollutants under different conditions

In brief, a suitable catalyst and PMS were immediately placed into a container containing rhodamine B (denoted as RhB) aqueous solution at room temperature. Afterward, the reaction was carried out. At appropriate time intervals, a suitable volume of the reaction solution was collected and methanol was added into the above-mentioned solution to capture reactive oxygen species. The absorbance of RhB was measured using a photometer. The pH was regulated with dilute H_2_SO_4_ or NaOH solution and measured using a pH meter before and after the reaction. According to the experimental requirements, the important reaction parameters and the related chemical reagents were timely adjusted. The quantification of PMS during degradation was measured by a potassium iodide (KI) method, and this is as follows: a 0.5 M KI solution was prepared, 1 mL reaction solution after the centrifugation was added into 1 mL of the KI solution, and finally, the mixture solution was further diluted with a suitable volume of distilled water. After 0.5 h, the absorbance of solution was measured at 351 nm.

## 3. Results and discussion

### 3.1. Characterization of different catalysts

**Fig. 1:**
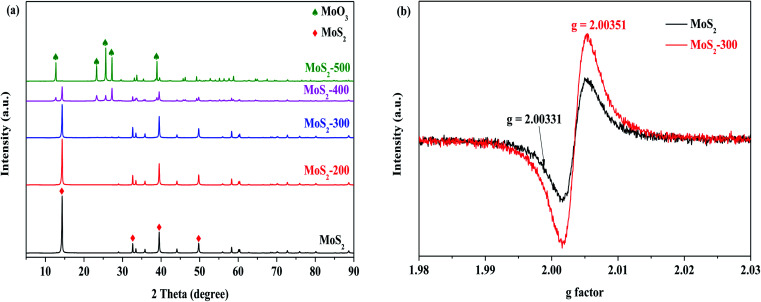
(a) XRD patterns of a series of MoS_2_ catalysts. (b) EPR signal of the sulfur vacancy of MoS_2_ and MoS_2_-300.

The XRD patterns of a series of MoS_2_ and MoS_2_-*X* (*X* = 200, 300, 400 and 500 °C) catalysts are displayed in Fig. 1 (a). It is observed that the diffraction peaks appeared at 2*θ* = 14.38°, 32.68°, 39.54°, 49.79° and 58.33°, which were attributed to the characteristic of MoS_2_ (PDF #37-1492). With increasing temperature below 400 °C, these samples possessed similar diffraction characteristic peaks as MoS_2_. This indicated that the structure of the treated catalyst remained unchanged relative to the untreated sample. However, the intensity of diffraction characteristic peaks in catalysts continued to reduce below 400 °C, as compared to that of MoS_2_. This is derived from the loss of the sulfur atoms in MoS_2_ at high temperatures, thereby resulting in a decrease in the crystallization of catalysts; in other words, sulfur vacancies may be formed. While the treatment temperature arrived at 400 °C, a great change occurred. The intensity of diffraction characteristic peaks for the MoS_2_-400 catalyst was remarkably reduced. Meanwhile, the diffraction characteristic peaks appeared at 2*θ* = 12.76°, 23.33°, 25.70°, 27.33°, 38.58° and 38.94°, which were ascribed to the characteristics of MoO_3_ (PDF #05-0508). The results indicated that the original structure of MoS_2_ was to some degree damaged after the treatment. This decreased degree became obvious with the further increase in temperature. To further explore the existence of the vacancy, the catalysts were characterized by EPR. The results indicated that the *g* value of MoS_2_-300 was slightly changed but the signal intensity of MoS_2_-300 was greatly altered relative to those of MoS_2_, as shown in Fig. 1(b). This suggested that the vacancies were indeed formed after the high-temperature treatment of the parent sample. This is in agreement with the results reported in the literature.^31^

**Fig. 2:**
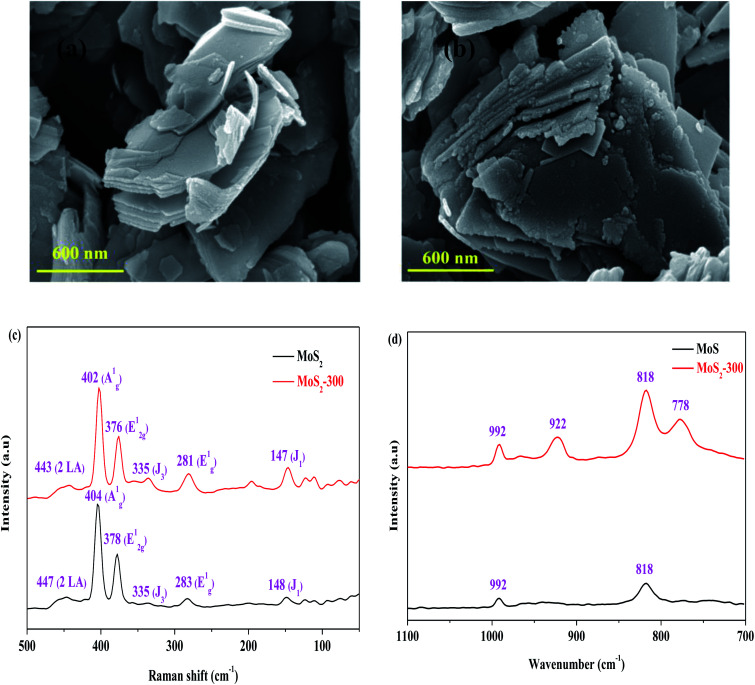
(a) SEM image of MoS_2_. (b) SEM image of MoS_2_-300. (c) Raman spectra of MoS_2_. (d) Raman spectra of MoS_2_-300.

Table 1 presents the results of pore structures of MoS_2_ and MoS_2_-300 catalysts. The *S*_BET_, *V*_total_ and *D*_A_ values of the MoS_2_ catalyst were 10.54 m^2^ g^−1^, 0.0360 cm^3^ g^−1^ and 13.64 nm, respectively. After the high-temperature treatment, the above-mentioned values were all reduced. It showed that the pore structure of MoS_2_ was to some extent destroyed. In combination with the results of RhB degradation, the reduction of *S*_BET_ did not affect the degradation of RhB. The SEM images of MoS_2_ and MoS_2_-300 catalysts are displayed. As observed in Fig. 2(a) and (b), MoS_2_ exhibited a smooth lamellar structure. After the treatment, the MoS_2_-300 still kept the lamellar structure but its surface became a little rough. This change is the main reason for the reduction in the *S*_BET_ value of the catalyst. The above-mentioned results further indicated that the treated catalyst maintained the original structure of MoS_2_.

**Table 1:** BET and pore information of different MoS_2_-based catalysts^a^

Catalysts	*S*_BET_ (m^2^ g^−1^)	*V*_total_ (cm^3^ g^−1^)	*D*_A_ (nm)
MoS_2_	10.54	0.0360	13.64
MoS_2_-300	8.95	0.0185	8.29

a *S*_BET_, *V*_total_ and *D*_A_ were respectively denoted as the specific surface area, total pore volume and total pore size of catalysts.

The Raman spectra of MoS_2_ and MoS_2_-300 catalysts were investigated, and the results are shown in Fig. 2(c) and (d). It is observed from Fig. 2(c) that the two samples exhibited similar spectra in the region of 100–500 cm^−1^. Generally, the typical spectra regions located at 376–378 cm^−1^, 402–405 cm^−1^ and 445–448 cm^−1^ were respectively assigned to the outer surface of Mo–S (E^1^_2g_), inner surface of Mo–S (A^1^_g_) and substitution acoustic (2 × LA) models.^33^ These models belonged to the typical characteristic structure of 2H-MoS_2_. It is the most stable structure of MoS_2_. In general, the E^1^_2g_ model was selectively excited toward the facial feature, while the A^1^_g_ model was preferentially excited to the corner feature arising from the polarization.^33^ In addition to E^1^_2g_, A^1^_g_ and 2 × LA, there were three additional models, namely, J_1_ (148 cm^−1^), E^1^_g_ (283 cm^−1^) and J_3_ (335 cm^−1^). J_1_ and J_3_ belonged to the super-lattice structure of 1T-MoS_2_, and E^1^_g_ belonged to the eight coordinated Mo atoms in 1T-MoS_2_. The results indicated that the 1T- and 2H-phases co-existed in MoS_2_, that is, it is one 1T–2H mixture structure. The peak at 992 cm^−1^ (2 × E^1^_2g_) belonged to the asymmetric stretching vibration, *v*_as_, of the Mo=O_(1)_ model.^33^ This may be due to the slight oxidation of the unsaturated Mo and S atoms or the trace incomplete reduction of Mo(vi) during the commercial synthesis of MoS_2_. Besides, the model (2 × A^1^_g_) appeared at 818 cm^−1^. After treatment at 300 °C, the positions of A^1^_g_, E^1^_2g_ and 2 × LA in the sample tended to be smaller wavenumbers, and the strength ratio of A^1^_g_ and E^1^_2g_ was decreased from 1.87 to 1.72, implying that part of the Mo–S bond was converted from outside to inside. The strength of 2 × E^1^_2g_ and 2 × A^1^_g_ in the MoS_2_-300 catalyst became stronger, hinting that more MoO_3_ species appeared on the surface of the catalyst due to the oxidation during heating treatment. Some new characteristic peaks such as 922 and 778 cm^−1^, were found in the MoS_2_-300 catalyst, implying the formation of a new Mo species. In a word, the above-mentioned results showed that the structure of the treated catalyst was partly altered.

**Fig. 3:**
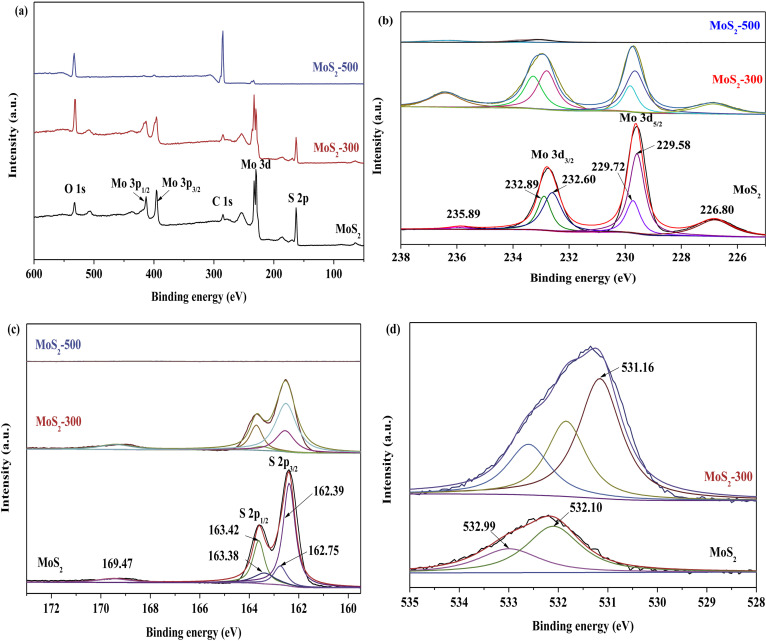
XPS spectra of (a) full-range scan of a series of MoS_2_ catalysts, (b) Mo 3d, (c) S 2p, and (d) O 1s.

Fig. 3 shows the XPS spectra of MoS_2_ and MoS_2_-*X* (*X*: 300 and 500 °C) catalysts, and some important results are also displayed in Table S1†. In the case of Mo 3d of MoS_2_, one characteristic peak with 226.80 eV of binding energy appeared, belonging to the sulfur atoms at the end of the Mo–S bond.^30–34^ The binding energies located at 229.60 and 232.75 eV corresponding to Mo 3d_5/2_ and Mo 3d_3/2_ were attributed to the characteristic peaks of Mo(iv).^32^ These peaks were further divided into four small peaks, corresponding to the characteristic phases of 2H-MoS_2_ (232.89 and 229.72 eV) and 1T-MoS_2_ (232.60 and 229.58 eV). Besides, one extremely weak characteristic peak was presented at 235.89 eV, which ascribed to the Mo-based oxides, in other words, it was attributed to the characteristic peaks of Mo(vi).^37^ As shown in Table S1†, the concentration of Mo(iv) and the ratio of Mo(iv) and Mo(vi) were both decreased after the treatment, especially at 500 °C. However, the ratio of 2H- and 1T-MoS_2_ first increased and then decreased. It hinted that Mo(iv) did not act as a main catalytic active site. In previous studies,^30,32–34^ most of the researchers considered that Mo(iv) was in favor of activating PMS. For S 2p of MoS_2_, there appeared two characteristic peaks located at 163.60 and 162.40 eV of binding energy, corresponding to S^2−^ 2p_1/2_ and S^2−^ 2p_3/2_. These peaks were subdivided into four small peaks, which were attributed to the characteristic phases of 2H-MoS_2_ (163.42 and 162.75 eV) and 1T-MoS_2_ (163.38 and 162.39 eV). Additionally, one very weak peak with 169.47 eV of binding energy was also observed. It was ascribed to the characteristic peak of S_I_ (–SO_*n*_–) species, derived from the oxidation of MoS_2_.^33^ With the increase in temperature, the concentration of S^2−^ species decreased, and even disappeared at 500 °C, whereas the concentration of S_I_ species was remarkably increased. This meant that the oxidation of catalysts was extremely serious. For O 1s of MoS_2_, there appeared two characteristic peaks located at 532.99 and 532.10 eV of binding energy, corresponding to adsorbed molecular water and surface-adsorbed oxygen species,^33^ respectively. After the treatment at 300 °C, there was an additional characteristic peak located at 531.16 eV, which was attributed to the oxygen coordination like oxygen vacancies.^33^ It further confirmed that oxygen atoms were introduced by replacing sulfur atoms after the treatment. In a word, the changes in MoS_2_ appeared after the high-temperature treatment, including the formation of new vacancies and the concentration of different MoS_2_ phases.

### 3.2. Removal of organic pollutants over various catalysts

#### 3.2.1. Removal of RhB in the catalytic systems

**Fig. 4:**
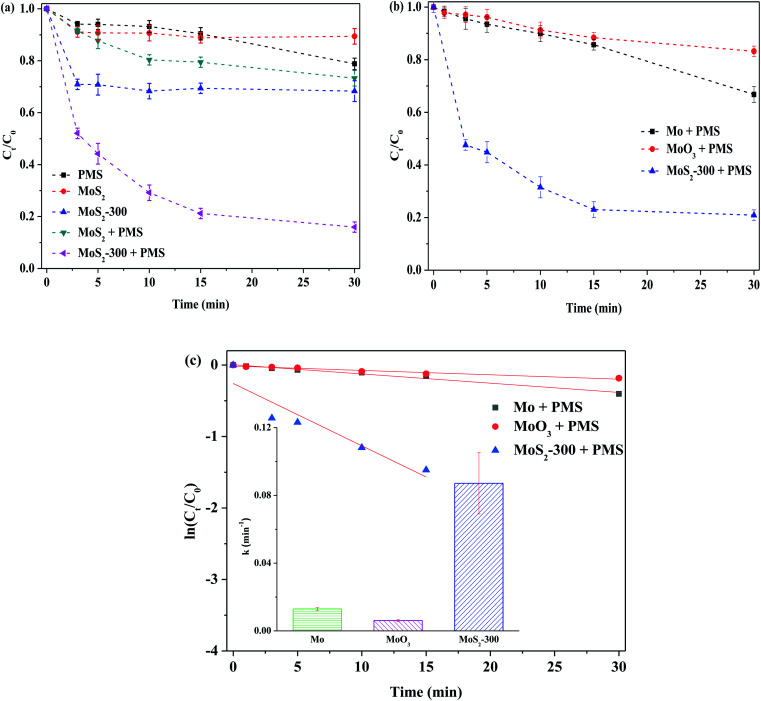
Degradation of 10 mg L^−1^ RhB in different process in the darkness. (a) Adsorption and oxidation of MoS_2_ and MoS_2_-300. Reaction conditions: initial pH = 3.0, [PMS] = 1.0 mM, [catal.] = 1.0 g L^−1^. (b) Oxidation of different Mo-based catalysts. (c) Reaction rate constant of different oxidation systems. Reaction conditions: initial pH = 3.0, [PMS] = 0.5 mM, [catal.] = 1.0 g L^−1^.

Fig. 4 displays the removal of RhB by PMS activated with different MoS_2_ catalysts. The degradation efficiency of RhB was only about 11% after 30 min of reaction, while MoS_2_ was employed. After PMS was added, the degradation efficiency of RhB was increased up to 27% at 30 min, hinting that the MoS_2_-activated PMS could promote the degradation of organic pollutants in solutions. This is consistent with the results reported in the literature.^30,32^ However, this improvement was limited. In order to further enhance the removal of RhB, MoS_2_-300 was selected as a heterogeneous catalyst to activate PMS. The degradation efficiency of RhB quickly reached *ca.* 84% at 30 min. In order to verify the role of MoS_2_-300, the adsorption experiment was carried out to confirm the contribution of adsorption in this reaction. The result indicated that the degradation efficiency of RhB was about 32% after 30 min of reaction, meaning that MoS_2_ with defect sites was beneficial for the removal of RhB. Du *et al.*^32^ found by using theoretical calculation (DFT) that the surface of MoS_2_ could strengthen the adsorbed PMS in the presence of sulfur vacancy, and meanwhile, the O–O bonds in PMS could be further extended to form reactive oxygen species. To demonstrate the roles of Mo and sulfur species, several Mo-based catalysts were used to activate PMS to degrade RhB (see Fig. 4(b)). As shown in this figure, while Mo or MoO_3_ acted as a heterogeneous catalyst, the degradation efficiency of RhB did not exceed 35%. According to the pseudo-first-order kinetics, the reaction rate constant (*k*) was calculated. As shown in Fig. 4(c), the *k* value of the MoS_2_-300/PMS system was remarkably higher than that of other reaction systems. The above-mentioned results indicated that the sulfur defect sites could indeed significantly strengthen the ability of degradation of RhB by activated PMS.

Fig. S2† compares the removal of RhB by MoS_2_-300-activated PMS with or without LED illumination. The degradation efficiency of RhB was about 84% at 30 min in the darkness. After the darkness was switched into LED illumination, the degradation efficiency of RhB was about 86%. It showed that the presence or absence of light illumination had little effect on the removal of RhB. Generally, the defect sites are classified into bulk and surface defect sites. For the photocatalytic reaction, the bulk defect sites often acted as the combination center of photogenerated hole–electron pairs (h^+^–e^−^), which had negative influence on the degradation reaction. In this study, MoS_2_-300 was used as a photocatalyst. Under LED illumination, h^+^–e^−^ was generated on the MoS_2_-300 catalyst, in which h^+^ could directly oxidize RhB, and meanwhile, e^−^ was transferred for PMS and/or dissolved oxygen in the solution to generate reactive oxygen species. The above-mentioned synergy could compensate for the negative effect from the defect sites. Hence, the presence or absence of LED illumination hardly affected the degradation of RhB in this reaction.

**Fig. 5:**
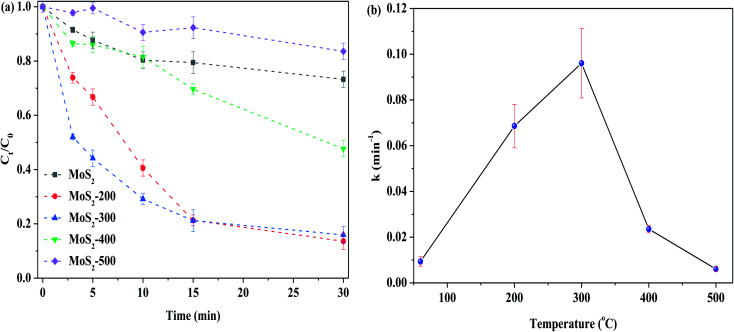
(a) Effect of the calcined temperature on the degradation of RhB. (b) Relationship between calcined temperature and reaction rate constant. Reaction conditions: [RhB] = 10 mg L^−1^, initial pH = 3.0, [PMS] = 1.0 mM, [catal.] = 1.0 g L^−1^, in the darkness.

Fig. 5(a) shows the removal of RhB by MoS_2_ and MoS_2_-*X* (*X*: 200, 300, 400 and 500 °C) at different temperatures activating PMS in the darkness. One can see that the degradation of RhB was low, while MoS_2_ was not treated by heat. With the increase in temperature, the degradation efficiency of RhB was increased. When the temperature arrived at 300 °C, the degradation efficiency of RhB reached the highest value. With the further increase in temperature, the degradation efficiency of RhB started to reduce. As depicted in Fig. 5(b), this change is well reflected on the basis of the relationship between treated temperature and *k*, showing that the reaction rate is closely related to the treatment temperature of the catalyst. This is because MoS_2_ is treated at high temperatures, resulting in the formation of defect sites, thus exposing more active sites. In this case, more reactive oxygen species are formed from PMS, which, in turn, enhance the degradation of RhB. The temperature is too high, in this case, the structure of MoS_2_ is destructed, as confirmed by XRD characterization (see Fig. 1(a)), and hence, it is disadvantageous for the activation of PMS. Normally, MoS_2_ began to be decomposed into MoO_3_ at 315 °C. However, the latter was less capable of activating PMS than the former, as evidenced from Fig. 4(b). Accordingly, the degradation efficiency of RhB was decreased, particularly the higher temperature. The degradation of RhB by PMS activated with MoS_2_-300 for different time periods in the darkness was studied, and the results are displayed in Fig. S3.† It was found that the time was in the range of 4–12 h, and the degradation efficiency of RhB over all these catalysts was maintained at 79–88% after the reaction. This meant that the time had little effect on the degradation of RhB.

**Fig. 6:**
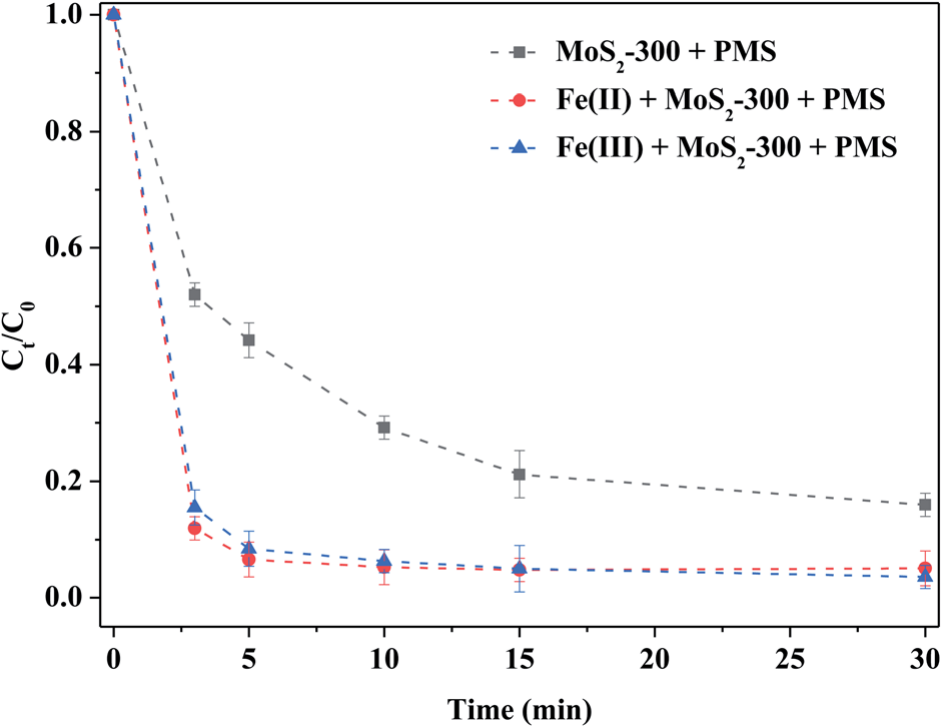
Comparison of homogeneous Fe ions activating PMS in the degradation of RhB. Reaction conditions: [RhB] = 10 mg L^−1^, initial pH = 3.0, [PMS] = 1.0 mM, [MoS_2_-300] = 1.0 g L^−1^, [Fe(ii)] = [Fe(iii)] = 0.20 mM, in the darkness.

Fig. 6 shows the degradation of RhB by MoS_2_-300-activated PMS with homogeneous Fe(ii) or Fe(iii) ions. Compared to the control experiment, Fe(ii) or Fe(iii) could strengthen the degradation ability of RhB by the MoS_2_-300/PMS system. For example, the degradation efficiency of RhB reached 90% in both the reaction systems after 5.0 min of reaction, which was higher than that of the control experiment. Kuang *et al.*^31^ pointed out that the Fe(iii) adsorption energy on MoS_2_ was −1.83 eV and −3.62 eV in the absence and presence of sulfur vacancies, respectively. That is to say, MoS_2_ with sulfur vacancies in thermodynamics was more favorable to adsorb Fe(iii), and thus, promoted the reaction between Fe(iii) and MoS_2_. As a result, the sulfur defect sites near MoS_2_-300 could accelerate the cycles of Fe(ii)/Fe(iii) to produce more reactive oxygen species, as shown in formulas (3-1)–(3-3).^31^ Consequently, Fe(ii) or Fe(iii) could boost the ability of removal of RhB by MoS_2_-300-activated PMS.

3-1 Mo(iv) + 2Fe(iii) → Mo(vi) + 2Fe(ii)

3-2 Mo(vi) + 2HSO_5_^−^ → Mo(iv) + 2SO_5_˙^−^ + 2H^+^

3-3 Fe(ii) + HSO_5_^−^ → Fe(iii) + OH^−^ + SO_4_˙^−^

Fig. S4† demonstrates the removal of RhB by different oxidants activated with MoS_2_-300. It is found that the degradation efficiency of RhB was about 80%, while PMS was used as an oxidant, and about 60% of RhB was obtained when PDS or H_2_O_2_ was employed as an oxidant. The above-mentioned results indicated that MoS_2_-300 has a strong activation ability toward typical oxidants. However, their differences were mainly due to the different properties of oxidants. The structure of PDS or H_2_O_2_ was symmetrical, whereas that of PMS was asymmetrical. In this case, PMS was easier to be activated than PDS and H_2_O_2_. In addition, the types of reactive oxygen species formed by PMS were different from those by PDS and H_2_O_2_ under the same conditions.^33,34^ For instances, while PMS was activated, the reactive oxygen species mainly included SO_4_˙^−^, ˙OH and ^1^O_2_. On the contrary, while PDS was activated, the main reactive oxygen species were ˙OH, O_2_˙^−^ and ^1^O_2_. In general, SO_4_˙^−^ had a higher catalytic oxidation capacity than that of ˙OH, O_2_˙^−^ and ^1^O_2_. Besides, the repulsion between MoS_2_ and the oxidant played an important role. The surface of MoS_2_ exhibited negative charges,^38^ and thus, the repulsive force between PDS (S_2_O_8_^2−^) and MoS_2_ became larger than that of PMS (HSO_5_^−^) with MoS_2_. As a result, the MoS_2_-300 catalyst-activated PMS possessed a higher degradation efficiency of RhB. Fig. S5† displays the removal of different organic dyes (including RhB, methyl blue (MB), methyl orange (MO) and orange II (AOII)) by MoS_2_-300 activating PMS in the darkness. Most of them were quickly degraded under the same reaction condition. It indicated that this catalytic system had a good removal ability for organic dyes in wastewater.

#### 3.2.2. Optimized reaction conditions

**Fig. 7:**
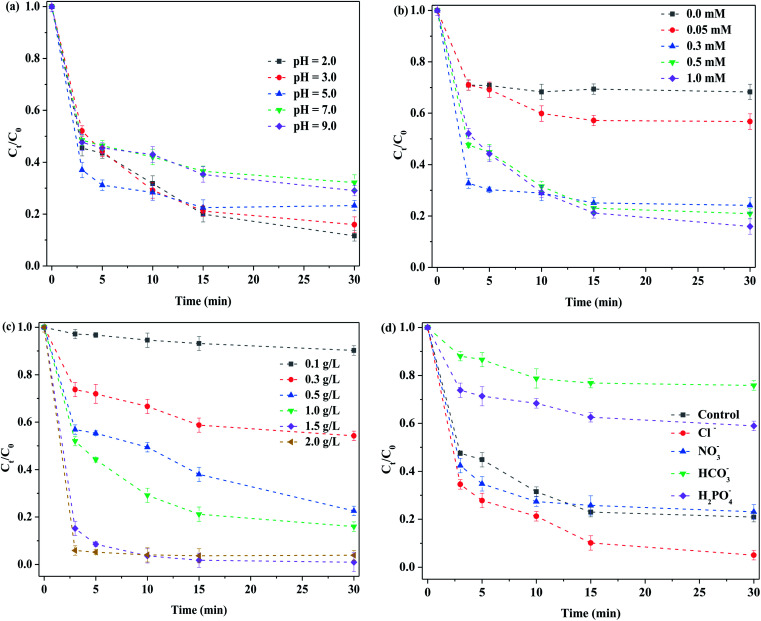
Influence of reaction conditions on the degradation of RhB: (a) initial pH; (b) PMS concentration; (c) catalyst concentration; and (d) anions. Reaction conditions: [RhB] = 10 mg L^−1^, [anions] = 100 mM, in the darkness.

Fig. 7(a) displays the removal of RhB by MoS_2_-300 activated at different initial pH values. Herein, no buffer solution was chosen, which avoided its interference. While the initial solution pH was 2.0, the degradation efficiency of RhB was about 88% at 30 min. The removal of RhB tended to decrease with the increase in the initial solution pH. In general, the degradation efficiency of RhB was maintained at 69–88% while the initial solution pH was in the range of 2.0–9.0. Obviously, the system possessed a wide pH range. The p*K*_a_ value of PMS was 9.3;^39^ in other words, while the solution pH was above 9.3, PMS started to convert into SO_5_^2−^. Clearly, the conversion of PMS was not one primary factor in the reduction degradation of RhB. While the acidic solution was used, the sulfur atoms on the corner of the MoS_2_ catalyst reacted with H^+^ in the solution, and therefore, the sulfur atoms were consumed.^40^ Concurrently, more sulfur vacancies were exposed, thereby accelerating the electron transfer between PMS and sulfur vacancies. Under alkaline conditions, the above-mentioned trapping reaction did not occur, thus preventing the exposure of sulfur vacancies. Therefore, the removal of RhB was decreased. Nevertheless, whether the initial reaction solution was acidic or alkaline, the final solution pH reached 2.0–3.5 after the reaction, as displayed in Fig. S6.† In this case, it was in favor of adsorbing RhB on the surface of the catalyst. It has been reported that the surface of MoS_2_ showed negative charges at pH = 2–9,^41^ and RhB was a cationic dye. Consequently, the MoS_2_-300 was favorable for the adsorption of RhB in a wide pH range. The roles of sulfur vacancies in MoS_2_-based catalysts after the high-temperature treatment and during the reaction were discussed. On the basis of the removal of RhB, the order of the catalytic activity is as follows: MoS_2_-300 > MoS_2_. The fact told that it was easier to activate PMS in the presence of vacancy in the catalyst, while the solution was in an acidic environment.

Fig. 7(b) demonstrates the effect of concentrations of PMS on the removal of RhB. While PMS was not added into the reaction solution, the degradation efficiency of RhB was about 32% in 30 min, which was mainly attributed to the adsorption of the catalyst. When the concentration of PMS was 0.05 mM, the degradation efficiency of RhB increased from 32% to 43%. It showed that MoS_2_-300 could activate PMS to form reactive oxygen species, which was beneficial for degrading RhB. The results meant that PMS itself had a limited ability to remove RhB. With the increase in the concentration of PMS, the removal of RhB was further increased. When 0.5 mM of PMS was employed, the degradation efficiency of RhB reached about 79%. By further increasing the concentration of PMS, the degradation efficiency of RhB was slightly increased up to 84%. More reactive oxygen species were generated with the increase in the concentration of PMS, and thus, the degradation efficiency of RhB was increased. The repulsive force between MoS_2_-300 and PMS was further enhanced, while the higher concentration of PMS was employed.^31^ Accordingly, the removal of RhB tended to be stable.

Fig. 7(c) displays the effect of dosages of the MoS_2_-300 catalyst on the removal of RhB. It was found that when the concentration of the catalyst was 0.1 g L^−1^, the degradation efficiency of RhB was only 9.8% at 30 min. With the increase in the dosage of the catalyst, the removal of RhB was increased. When the dosage of the catalyst arrived at 1.5 g L^−1^, the degradation efficiency of RhB was over 90% only for 5 min. With the further increase in the dosage of the catalyst, the removal of RhB became stable. The dosage of the catalyst was insufficient, and there were no enough active sites for activating PMS, and thus the removal of RhB was low. With the increase in the dosage of the catalyst, PMS activated by the catalyst produced more reactive oxygen species. Accordingly, the removal of RhB was increased. While the dosage of the catalyst was too high, the concentration of active sites was reduced due to the collision between catalyst particles. Moreover, a limited reactive oxygen species were formed because the PMS concentration was constant. Accordingly, the removal of RhB retained a little change.

Fig. 7(d) displays the influence of various anions on the removal of RhB. The degradation efficiency of RhB was about 80% without the addition of extra anions, while the reaction time was 30 min. While different anions were respectively added, they had different effects on the removal of RhB. For examples, after the addition of Cl^−^, the removal of RhB was further increased, probably due to the formation of more active species such as Cl˙ in the reaction system,^42^ as observed in formula (3-4). After the addition of NO_3_^−^, the removal of RhB was slightly decreased, which may be attributed to the formation of less reactive oxygen species, as shown in formula (3-5). Similar results were found.^30^ From this point of view, it is concluded that the main reactive oxygen species in this reaction system were not typical strong oxidizing species such as SO_4_˙^−^ and ˙OH. However, when HCO_3_^−^ or H_2_PO_4_^−^ was added into the initial solution, the removal of RhB was inhibited in both cases, especially for HCO_3_^−^. For instance, for HCO_3_^−^, the degradation efficiency of RhB was only about 20% after 60 min of reaction. When the pH of the solution was increased to 8–9, HCO_3_^−^ could neutralize most of H^+^ in the solution. As a result, the sulfur atoms in the corner of MoS_2_ were not captured. Therefore, the interaction between sulfur vacancies and PMS was significantly inhibited. For H_2_PO_4_^−^, the degradation efficiency of RhB was about 40% after 60 min of reaction. On the one hand, when H_2_PO_4_^−^ was added, the pH of the solution was increased and it affected the binding of sulfur atoms on the edge of MoS_2_ by H^+^. On the other hand, the phosphate in H_2_PO_4_^−^ could coordinate closely with Mo species on the MoS_2_ to form phosphomolybdate.^39^ In this case, it induced the steric hindrance effect and thus affected the activation of PMS. Accordingly, the removal of RhB was reduced, as compared to the control experiment.

3-4 Cl^−^ + SO_4_˙^−^ → Cl˙^−^ + SO_4_^2−^

3-5 NO_3_^−^ + SO_4_˙^−^ → NO_3_˙^−^ + SO_4_^2−^

#### 3.2.3. Stability of the catalyst

**Fig. 8:**
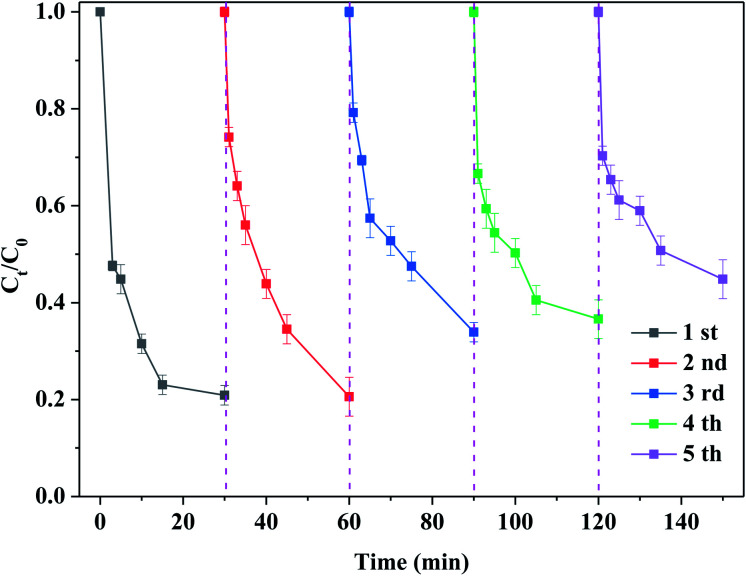
Stability of the catalyst. Reaction conditions: [RhB] = 10 mg L^−1^, initial pH = 3.0, [PMS] = 0.5 mM, [MoS_2_-300] = 1.0 g L^−1^, in the darkness.

Fig. 8 shows the stability of the catalyst. One can see that the degradation efficiency of RhB was about 80% after the first reaction. After the reaction, the catalyst was regenerated, that is, the reaction solution was filtered to achieve solid–liquid separation and then dried. Finally, it was calcined at 300 °C. The regenerated catalyst was added according to the reaction condition of the first reaction and then the second reaction was conducted. Under the same reaction condition, the degradation efficiency of RhB was about 80% after 30 min of the reaction. The above-mentioned steps were followed by the multiple reaction and regeneration. After the fifth reaction, the degradation efficiency of RhB still reached about 70% of the initial degradation efficiency. This indicated that the life of catalyst was long. It is noteworthy that the catalyst used in this experiment had to be treated at 300 °C. Under identical reaction conditions, the degradation efficiency of RhB was about 10% while the used catalyst after the first reaction was not calcined, as shown in Fig. S7(a).† Obviously, the reason of the deactivation of catalyst was to a great extent attributed to the covered and/or blocked by the intermediates or products. Besides, some sulfur vacancies were newly formed after the regeneration. Du *et al.*^32^ also found that the used MoS_2_ catalyst needed to be regenerated by H_2_O_2_ and the activity of the catalyst was almost recovered. It should be noted that the oxidation of MoS_2_ became increasingly serious during the reaction and regeneration. In this case, its structure and catalytic activity are difficult to fully be recovered, as evidenced in Fig. S2.† In this study, the initial catalytic activity could be restored by simple calcination without additional chemical treatment. Therefore, the regeneration used in this work had many advantages such as simplicity, safety and easy operation. After the reaction, the used catalyst was characterized by XRD and SEM techniques. As displayed in Fig. S7(b) and (c),† the phase and morphology of catalyst are basically the same before and after the reaction, suggesting that the structure of catalyst remained unchanged. After the reaction, the loss components of the catalyst were detected by the ICP-MS. It is observed from Table S2† that the concentrations of Mo and S elements in the solution were 36.2 and 91.6 mg L^−1^ after the reaction, respectively. Combined with the catalyst dosage, the loss efficiency of catalyst was 12.8%. This further confirmed that the catalyst possessed a good stability.

#### 3.2.4. Identified reactive oxygen species on the removal of RhB

**Fig. 9:**
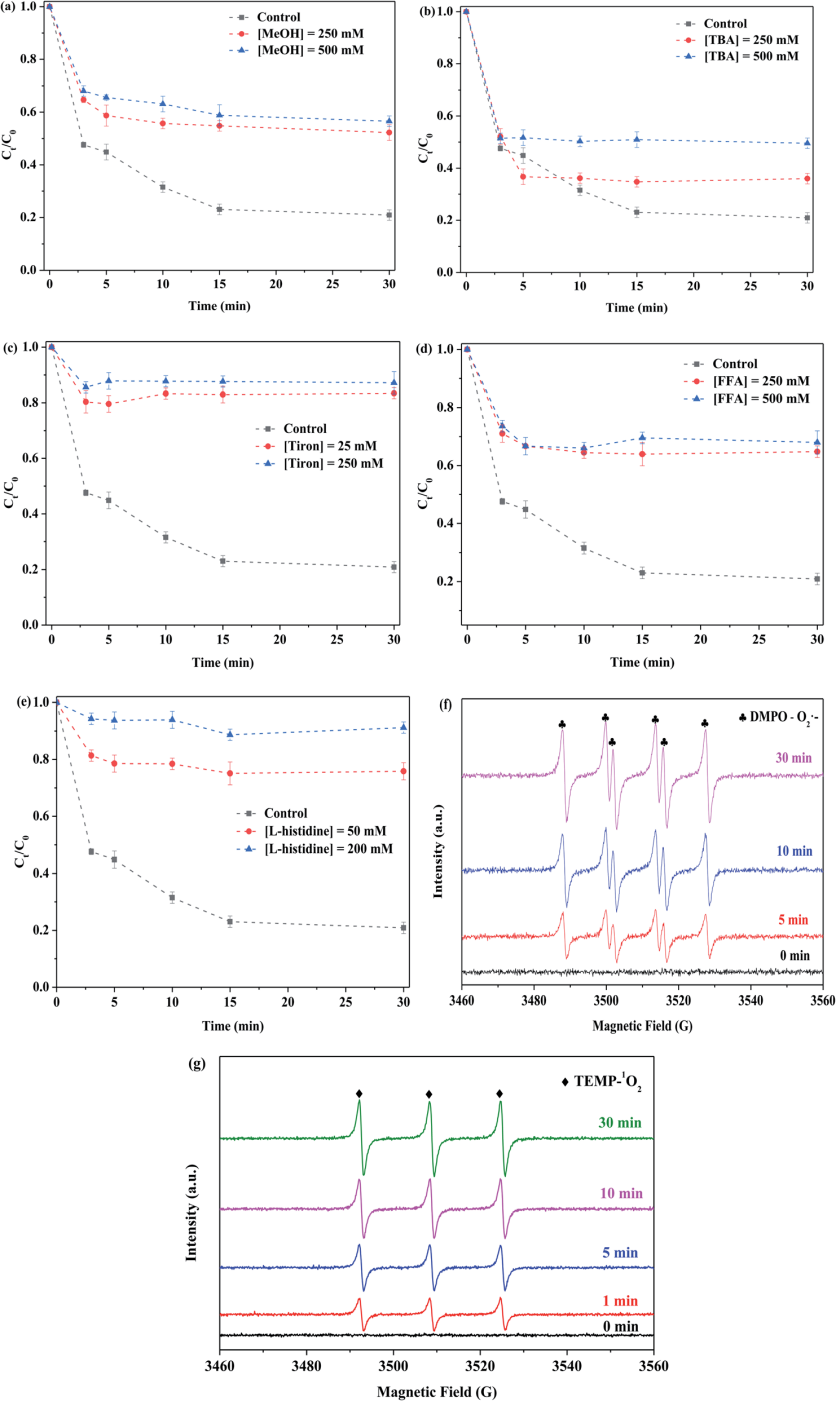
Effect of quenchers (a)–(e). (f) and (g) EPR signal of the PMS activation system for the detection of O_2_˙^−^ and ^1^O_2_ in the presence of DMPO and TEMP, respectively. Reaction conditions: [RhB] = 10 mg L^−1^, initial pH = 3.0, [PMS] = 0.5 mM, [MoS_2_-300] = 1.0 g L^−1^, in the darkness.

Fig. 9(a) displays the influence of methanol on the removal of RhB. Methanol (MeOH) could capture the SO_4_˙^−^ and ˙OH species, as there is no significant difference in reaction rate constants.^43^ In addition, *tert*-butanol (TBA) was able to identify ˙OH species.^31^ While methanol with different concentrations (250 and 500 mM) was respectively employed in this system, the degradation efficiency of RhB was decreased from 79% to 48% and 43%. This implied the presence of SO_4_˙^−^ and ˙OH in this reaction, and they contributed toward the removal of RhB. As shown in Fig. 9(b), while *tert*-butanol with different concentrations (250 and 500 mM) was used in the reaction solution, the degradation efficiency of RhB was decreased from 79% to 64% and 50%, respectively. It showed the existence of ˙OH in this reaction. Compared to SO_4_˙^−^, ˙OH played a greater role in the RhB degradation. The formation of SO_4_˙^−^ species was derived from the interaction of Mo(iv) with PMS, whereas the ˙OH species originated from the reaction of SO_4_˙^−^ with H_2_O, as shown in formulas (3-6) and (3-7):

3-6 Mo(iv) + HSO_5_^−^ → Mo(vi) + SO_4_˙^−^ + OH^−^

3-7 SO_4_˙^−^ + H_2_O → ˙OH + SO_4_^2−^ + H^+^

The influence of tiron on the RhB degradation was studied, and the results are shown in Fig. 9(c). The degradation efficiency of RhB quickly decreased from 79% to 17% and 13%, while tiron (25 and 250 mM) with different concentrations was respectively employed in the reaction solution. It showed that the reaction system contained a large number of O_2_˙^−^ species, and meanwhile, it played an important role in the removal of RhB. The MoS_2_-300 catalyst was rich in sulfur defect sites. These vacancies had a good affinity for PMS in water and then could react with each other to generate reactive oxygen species.^33^ At the liquid–solid interface, the intermediates were finally evolved into O_2_˙^−^, as shown in formulas (3-8) and (3-9):3-8
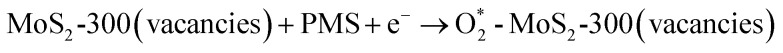
3-9
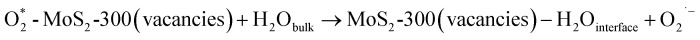


The effect of furfuryl alcohol on the RhB degradation was investigated. As shown in Fig. 9(d), furfuryl alcohol (FFA) with different concentrations (250 and 500 mM) was respectively used, and the degradation efficiency of RhB was reduced from 79% to 35% and 32%. Additionally, while l-histidine with different concentrations (50 and 200 mM) was employed in the reaction system, the degradation of RhB was also obviously decreased, as evidenced in Fig. 9(e). This indicated that the ^1^O_2_ species existed in this reaction system and played an important role in the degradation of organic pollutants. There were several formed sources of ^1^O_2_, and they are as follows: (i) O_2_˙^−^ as an intermediate,^33,43^ as shown in formulas (3-10) and (3-11); (ii) the vacancies interacted with PMS;^43^ and (iii) O_2_˙^−^ reduced Mo(vi),^44^ as shown in formulas (3-12):

3-10 2O_2_˙^−^ + H_2_O → H_2_O_2_ + OH^−^ + ^1^O_2_

3-11 O_2_˙^−^ +˙OH + 2H^+^ → OH^−^ + ^1^O_2_

3-12 Mo(vi) + O_2_˙^−^ + 2H^+^ → Mo(iv) + ^1^O_2_

In order to confirm the presence of O_2_˙^−^ and ^1^O_2_ in the reaction system, EPR was used to probe the generation of reactive oxygen species. DMPO (methanol as a solvent) was employed to detect the existence of O_2_˙^−^, and TEMP was applied for capturing ^1^O_2_. The EPR spectrum shown in Fig. 9(f) displays four big peaks and two small peaks for DMPO–O_2_˙^−^ spin adducts, and the intensity of the relative signals became stronger with the increase in reaction time. Similarly, the remarkable signal for the TEMPO was also found and the corresponding intensity was increased with the prolonged reaction time, as shown in Fig. 9(g). The results indicated that the reaction system contained O_2_˙^−^ and ^1^O_2_.

In a word, the reaction system included free radical species such as SO_4_˙^−^, ˙OH and O_2_˙^−^, and non-radical species like ^1^O_2_. Among them, O_2_˙^−^ and ^1^O_2_ were the main reactive oxygen species, as demonstrated in formula (3-13). In order to verify the source of these species, the variation in the PMS concentration before and after the reaction was investigated, and the results are shown in Fig. S8.† It was found that the decomposition efficiency of PMS reached 100% after the reaction. This indicated that the catalyst had a good activation ability toward PMS, and the source of reactive oxygen species was mainly the decomposition of PMS in this reaction.

3-13 RhB + SO_4_˙^−^/˙OH/O_2_˙^−^/^1^O_2_ → intermediates + CO_2_ + H_2_O

## 4. Conclusion

In summary, MoS_2_ with defect sites has been synthesized by high-temperature treatment. The temperature affected the change in MoS_2_ phases. It could promote the removal of RhB by activating PMS due to the existence of sulfur vacancies. In a wide range of initial pH, the catalytic system maintained a good degradation capacity. In addition, the foreign anions had different influences on the removal of RhB. Among them, Cl^−^ promoted the degradation reaction, whereas HCO_3_^−^ suppressed the removal of RhB. After running several reaction and regeneration cycles, the catalyst still retained good stability. It is confirmed that O_2_˙^−^ and ^1^O_2_ were the main active oxygen species in this reaction. It offered a new reaction pathway for the removal of organic pollutants in wastewater.

## Conflicts of interest

There are no conflicts to declare.

## Supplementary Material

RA-012-D2RA02448A-s001
