# Supplementary material for: Sulfur vacancies on MoS2 enhanced the activation of peroxymonosulfate through the co-existence of radical and non-radical pathways to degrade organic pollutants in wastewater
Source: RSC Adv. 2022 Sep 20;12(39):25364–76. doi: 10.1039/d2ra02448a (PMC9486530; doi:10.1039/d2ra02448a)
Supplement: RA-012-D2RA02448A-s001 [file RA-012-D2RA02448A-s001.pdf]

Electronic Supplementary Material (ESI) for RSC Advances.
This journal is © The Royal Society of Chemistry 2022

Support Materials

Sulfur Vacancy on MoS_2 Enhanced the Activation of Peroxymonosulfate through the Co-existence of Radical and Non-radical Pathways to Degrade Organic Pollutants in Wastewater

Cai-Wu Luo [a, b, c, *], Lei Cai [c], Chao Xie [c], Gang Li [a], Tian-Jiao Jiang [c]

[a] State Key Laboratory of Safety and Health for Metal Mines, Sinosteel Maanshan General Institute of Mining Research Co., Ltd, 243000, China

[b] Fujian Provincial Key Lab of Coastal Basin Environment, Fujian Polytechnic Normal University, 350300, China

[c] School of Resource Environmental and Safety Engineering, University of South China, 421000, China

* Corresponding author: Cai-Wu Luo;

Tel: +86-734-8282345;

E-mail addresses: luocaiwu00@126.com.

Figure S1

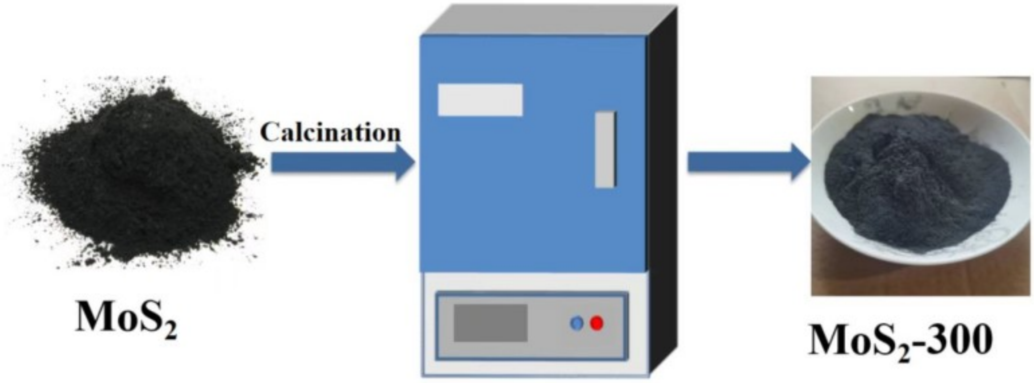

Figure S1 Preparation step of MoS_2-300 catalyst.

Figure S2

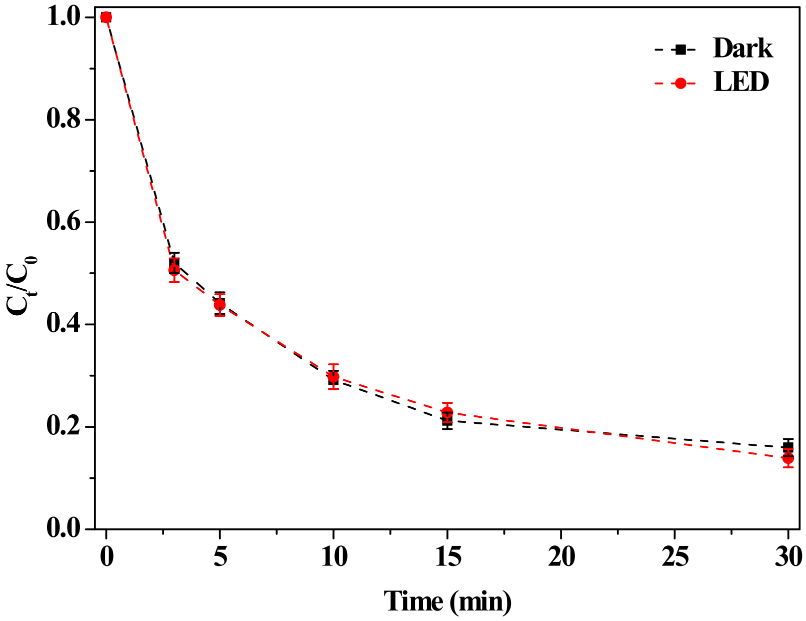

Figure S2 Comparison of darkness and LED illumination on degradation of RhB. Reaction conditions: [RhB] = 10 mg/L, [PMS] = 1.0 mM, [MoS_2-300] = 1.0 g/L, initial pH = 3.0.

Figure S3

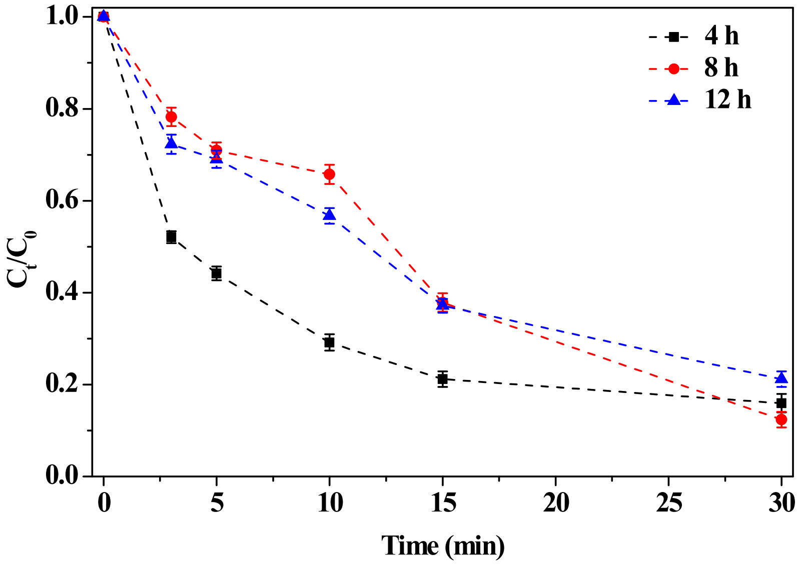

Figure S3 Effect of the treated time on the degradation of RhB. Reaction conditions: [RhB] = 10 mg/L, [PMS] = 1.0 mM, [MoS_2-300] = 1.0 g/L, initial pH = 3.0, in the darkness.

Figure S4

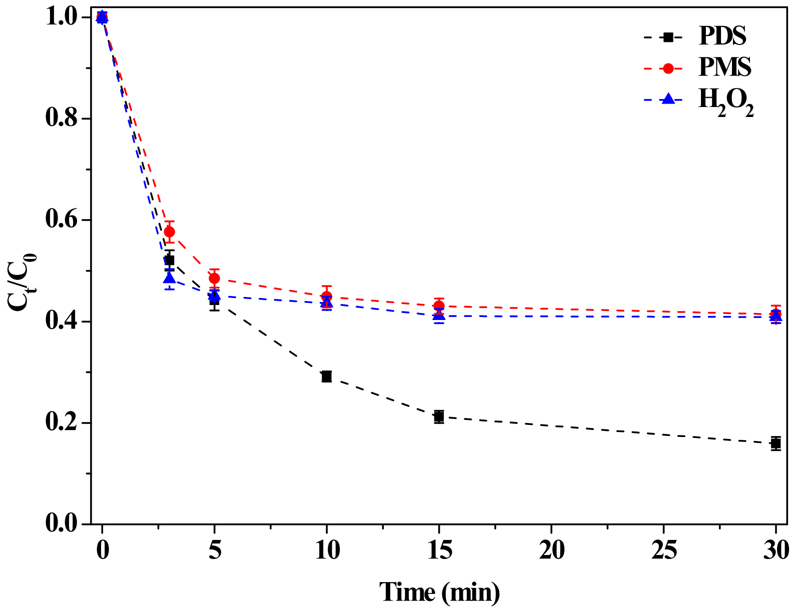

Figure S4 Removal of RhB with different oxidation system. Reaction conditions: [RhB] = 10 mg/L, [Oxidants] = 1.0 mM, [MoS_2-300] = 1.0 g/L, initial pH = 3.0, in the darkness.

Figure S5

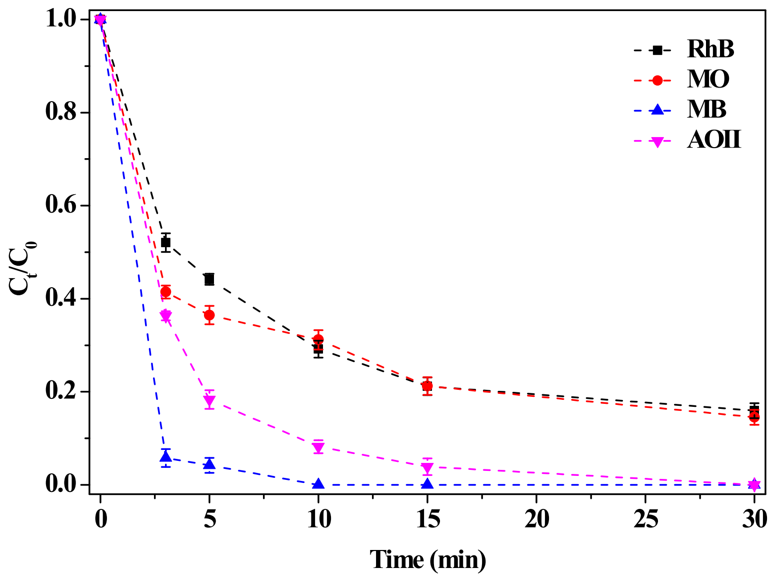

Figure S5 Removal of organic pollutants. Reaction conditions: [Pollutants] = 10 mg/L, [MoS_2-300] = 1.0 g/L, [PMS] = 1.0 mM, initial pH = 3.0, in the darkness.

Figure S6

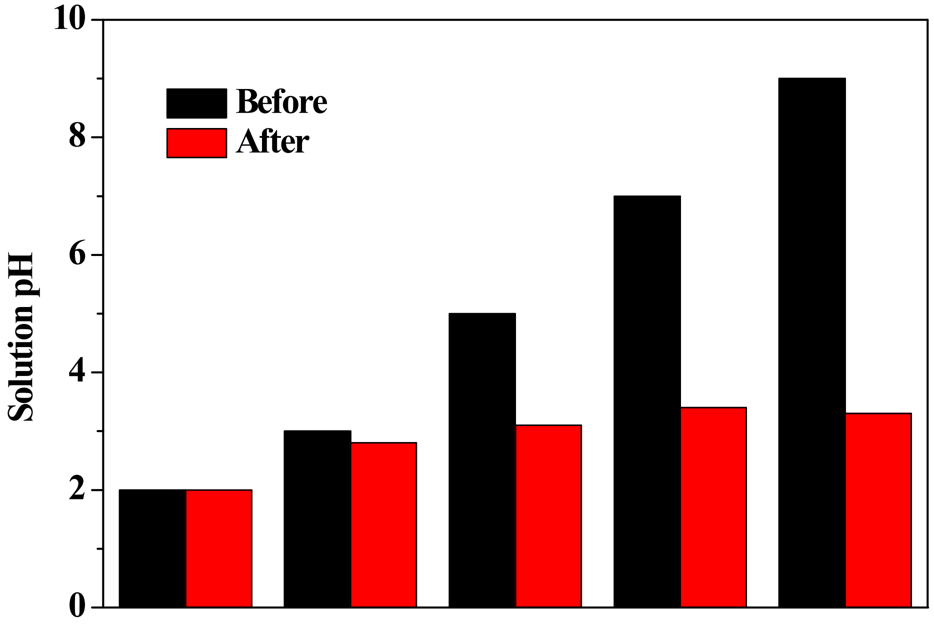

Figure S6 Changes of solution pH before and after the reaction.

Figure S7

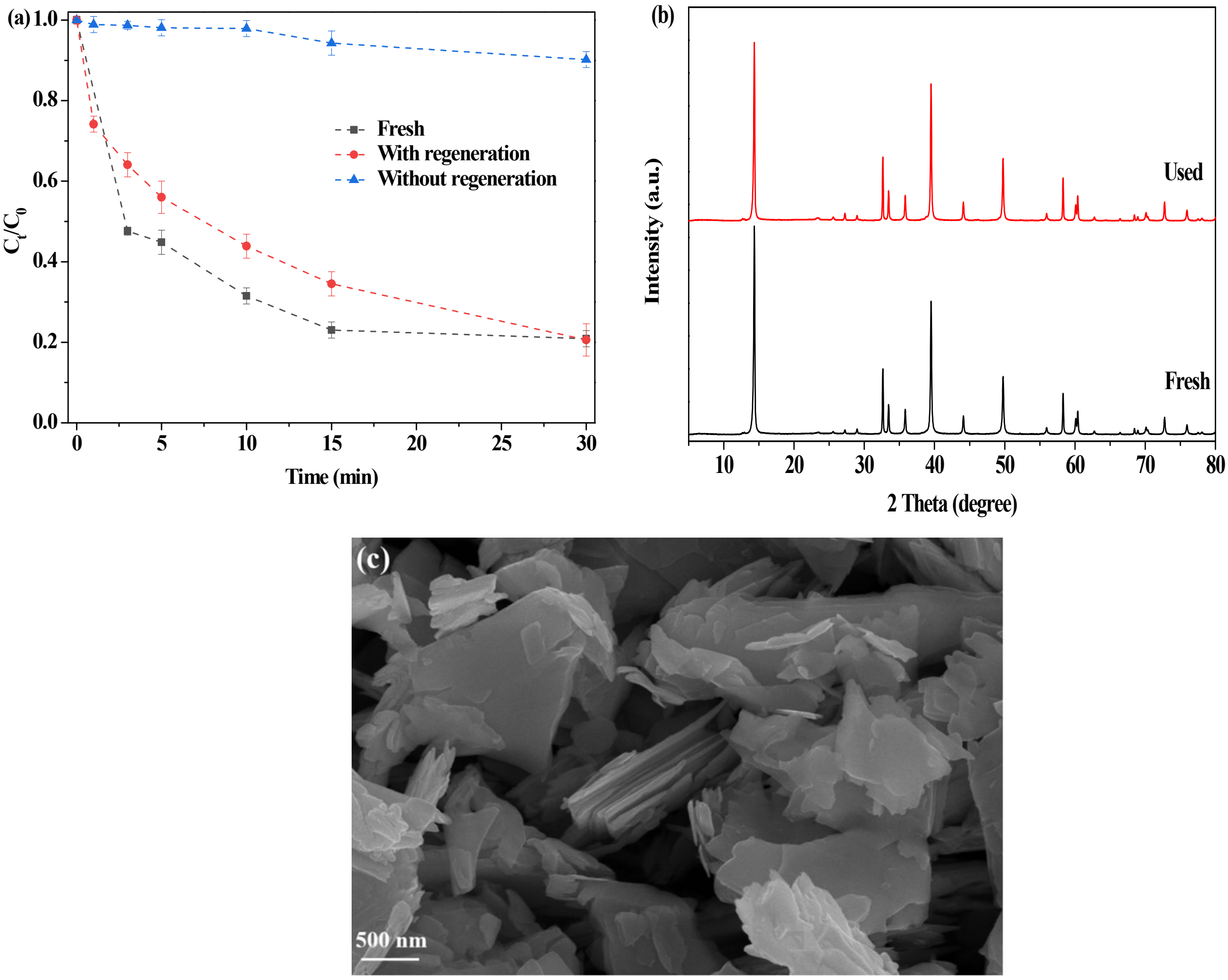

Figure S7 (a) Removal of RhB without or with regeneration; (b) XRD patterns of fresh and used MoS_2 catalysts; (c) SEM image of used MoS_2-300 catalyst. Reaction conditions: [RhB] = 10 mg/L, [PMS] = 0.5 mM, [MoS_2-300] = 1.0 g/L, initial pH = 3.0, in the darkness.

Figure S8

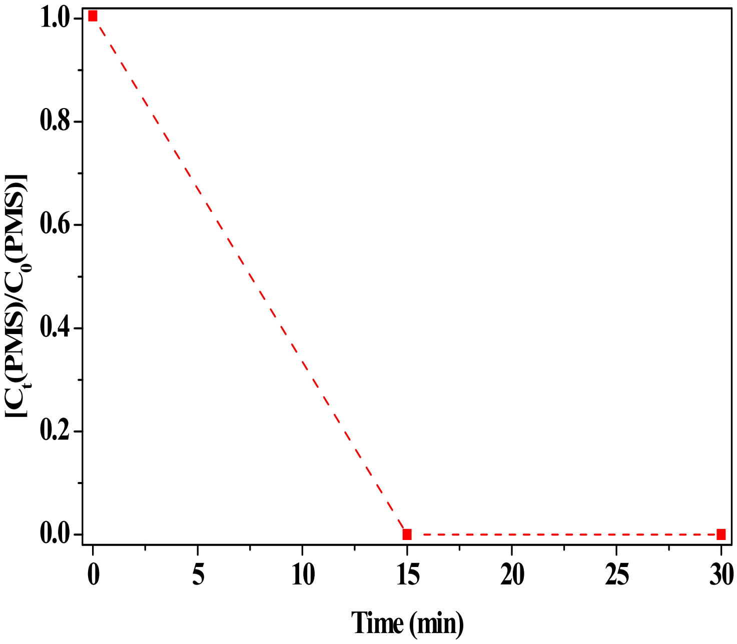

Figure S8 Decomposition of PMS. Reaction conditions: [RhB] = 10 mg/L, [MoS_2-300] = 1.0 g/L, [PMS] = 1.0 mM, initial pH = 3.0, in the darkness.

Table S1 Changes of surface Mo(IV) concentration, ratios of Mo(IV) and Mo(VI), and 2H-MoS_2 and 1T-MoS_2 phases in catalysts with different temperatures by XPS characterization [a].

Catalysts	Mo(IV)	Mo(IV)/Mo(VI)	2H-MoS_2/1T-MoS_2 [b]	2H-MoS_2/1T-MoS_2 [c]
MoS_2	98.3%	57.8	0.48	0.50
MoS_2-300	85.1%	5.7	0.62	0.66
MoS_2-500	58.8%	1.4	0.28	no

[a]: Based on the peak area of each component; [b]: Mo 3d; [c]: S 2p.

Table S2 The concentrations of detected elements in reaction solution after the fifth reaction by ICP-MS.

Catalysts	Mo (mg/L)	S (mg/L)
MoS_2-300	36.2	91.6
